# Change in walking for transport: a longitudinal study of the influence of neighbourhood disadvantage and individual-level socioeconomic position in mid-aged adults

**DOI:** 10.1186/s12966-014-0151-7

**Published:** 2014-12-10

**Authors:** Gavin Turrell, Belinda Hewitt, Michele Haynes, Andrea Nathan, Billie Giles-Corti

**Affiliations:** School of Public Health and Social Work, Queensland University of Technology, Brisbane, Queensland Australia; Institute for Social Science Research and School of Social Science, The University of Queensland, Brisbane, St Lucia, Queensland Australia; Institute for Social Science Research, The University of Queensland, Brisbane, St Lucia, Queensland Australia; McCaughey VicHealth Centre for Community Wellbeing, Melbourne School of Population and Global Health, The University of Melbourne, Melbourne, Victoria Australia

**Keywords:** Longitudinal, Walking, Active transport, Neighbourhood, Socioeconomic position, Ageing

## Abstract

**Background:**

Unlike leisure time physical activity, knowledge of the socioeconomic determinants of active transport is limited, research on this topic has produced mixed and inconsistent findings, and it remains unknown if peoples’ engagement in active transport declines as they age. This longitudinal study examined relationships between neighbourhood disadvantage, individual-level socioeconomic position and walking for transport (WfT) during mid- and early old-age (40 – 70 years). Three questions were addressed: (i) which socioeconomic groups walk for transport, (ii) does the amount of walking change over time as people age, and (iii) is the change socioeconomically patterned?

**Methods:**

The data come from the HABITAT study of physical activity, a bi-annual multilevel longitudinal survey of 11,036 residents of 200 neighbourhoods in Brisbane, Australia. At each wave (2007, 2009 and 2011) respondents estimated the duration (minutes) of WfT in the previous 7 days. Neighbourhood disadvantage was measured using a census-derived index comprising 17 different socioeconomic components, and individual-level socioeconomic position was measured using education, occupation, and household income. The data were analysed using multilevel mixed-effects logistic and linear regression.

**Results:**

The odds of being defined as a ‘never walker’ were significantly lower for residents of disadvantaged neighbourhoods, but significantly higher for the less educated, blue collar employees, and members of lower income households. WfT declined significantly over time as people aged and the declines were more precipitous for older persons. Average minutes of WfT declined for all neighbourhoods and most socioeconomic groups; however, the declines were steeper for the retired and members of low income households.

**Conclusions:**

Designing age-friendly neighbourhoods might slow or delay age-related declines in WfT and should be a priority. Steeper declines in WfT among residents of low income households may reflect their poorer health status and the impact of adverse socioeconomic exposures over the life course. Each of these declines represents a significant challenge to public health advocates, urban designers, and planners in their attempts to keep people active and healthy in their later years of life.

## Background

Ageing is associated with declines in physiologic, cardiovascular, and homeostatic reserve [1,2], declines in muscular strength, balance, and flexibility [3,4], and increases in overweight and obesity [5,6] and chronic disease [7,8]. Experimental, intervention, and epidemiological studies show that these declines in health and function can be prevented, slowed, or delayed by regular moderate-intensity physical activity (PA) [9-11]. Studies also show however that PA declines with age [12,13] and that sedentary behaviour increases [14]. Moreover, the range and diversity of PA narrows as we age and walking becomes the predominant activity [12,15]. Age-related declines in PA are not uniform within the population; rather, the declines are characterised by heterogeneity in terms of when in the life-course they commence and the steepness of their gradient [16,17]. Socioeconomic factors are key contributors to this heterogeneity. Whilst all people, irrespective of their socioeconomic circumstances inevitably undergo declines in PA as a result of physiologic limitations imposed by biological ageing [18], the declines are typically steeper for residents of disadvantaged neighbourhoods [19] and persons of low socioeconomic position (SEP)[20,21]. Given the strong (causal) link between PA and health [12] these steeper declines probably contribute to the poorer physical functioning [22,23] and higher levels of morbidity and mortality for chronic disease experienced by socioeconomically disadvantaged groups in mid-life and early old-age [24].

There are four primary domains of activity that contribute to total PA, and by extension, to energy expenditure, health, and well-being: leisure-time PA (LTPA), active transport (AT), and occupation and domestic related activity [25,26]. The vast majority of studies examining relationships between neighbourhood disadvantage, individual-level SEP and PA have focused on LTPA [27]. This research consistently shows that residents of advantaged neighbourhoods and persons of higher SEP (typically measured using education, occupation, and income) are more likely to participate in LTPA [28-30] and engage at a level that is sufficient for the accrual of health benefits [31]. By contrast, relationships between neighbourhood disadvantage, individual-level SEP and the other domains of PA have received limited attention. Arguably, a more complete understanding of the socioeconomic determinants of PA (and health) necessitates a broader conceptualisation of activity to include the other domains [32].

In this paper we focus on active transport (AT) and in particular walking for transport (WfT). AT is usually defined as ‘walking and cycling to get to and from places’ [33,34]. Increasingly, these modes of transport are being recognised as a way of incorporating PA into everyday life [35-37] and as a means by which physically inactive population groups (e.g. low SEP) can meet PA guidelines [38]. Importantly, research suggests that AT accrues health benefits independent of LTPA [39]. High levels of AT have been associated with lower all-cause mortality [40], a reduced risk of type 2 diabetes [41] and cardiovascular disease [42,43], and lower odds of overweight and obesity [44,45]. Unlike LTPA, our understanding of the association between neighbourhood disadvantage, individual-level SEP and AT is at a nascent stage: only a small number of studies have examined these relationships with inconsistent and contradictory findings [27].

We reviewed fourteen studies from developed countries that investigated the socioeconomic determinants of WfT. The review was limited to research that focused explicitly on transport-related walking and excluded those that used a measure of transport activity that combined both walking and cycling: these two behaviours often show opposite socioeconomic effects [33,38,46] hence these studies are likely to produce associations that are biased towards the null. In contrast to LTPA, research examining relationships between neighbourhood disadvantage, individual-level SEP and WfT has generated highly mixed findings, and consequentially, is complex and difficult to summarise in a straightforward manner. Similar observations have been made by others [33,47]. Specifically, the direction and strength of associations between the socioeconomic predictors used and WfT varies depending on the level of socioeconomic measurement (i.e. neighbourhood or individual), the type of individual-level socioeconomic indicator (e.g. education or income), how WfT is measured (e.g. frequency of walking or minutes walked), and the context in which walking is undertaken (e.g. walking to work, or to public transport). Thus whilst the evidence suggests that WfT is socioeconomically patterned in ways that are often distinct from LTPA, we are still some-way from having a clear picture of how commonly-used socioeconomic measures in PA research relate to WfT. Moreover, no longitudinal studies of neighbourhood disadvantage, SEP and WfT were found, hence it remains unknown if WfT declines over time, and whether, like LTPA, the declines are steeper for socioeconomically disadvantaged groups.

This paper addresses a number of these gaps and examines relationships between neighbourhood disadvantage, education, occupation, household income, and WfT, and how these relationships change over time. Three questions are addressed: (i) which socioeconomic groups walk for transport, (ii) does the amount of WfT change as people age, and (iii) is the change socioeconomically patterned? This investigation uses three waves of data from the HABITAT (**H**ow **A**reas in **B**risbane **I**nfluence Heal**T**h and **A**c**T**ivity) study. HABITAT is a multilevel longitudinal study of PA among mid-aged adults living in Brisbane, Australia [48,49]. The primary aim of HABITAT is to examine patterns of change in PA, sedentary behaviour, active transport and health between 2007 and 2018 and to assess the relative contributions of environmental, social, psychological and socio-demographic factors, to these changes.

## Methods

The HABITAT study received ethical clearance from the Queensland University of Technology Human Research Ethics Committee (Ref. Nos. 3967H & 1300000161).

### Sample design

Details about HABITAT’s baseline sampling design have been published elsewhere [48]. Briefly, a multi-stage probability sampling design was used to select a stratified random sample (m = 200) of Census Collector’s Districts (CCD), and from within each CCD, a random sample of people (on average 85 per CCD) aged 40–65 years.

### Neighbourhood-level unit of analysis, data sources, and measures

The primary area-level unit-of-analysis for the HABITAT study is the CCD: at the time the study commenced these were the smallest administrative units used by the Australian Bureau of Statistics (ABS) to collect census data. In urban areas such as Brisbane, a CCD contains an average of 200 private dwellings which are deemed to be relatively homogeneous in terms of their socioeconomic characteristics. CCDs are embedded within a larger suburb, hence the area corresponding to, and immediately surrounding, a CCD is likely to have meaning and significance for their residents: for this reason, we hereafter use the term ‘neighbourhood’ to refer to CCDs. Each of the 200 CCDs was assigned a socioeconomic score using the ABS’ Index of Relative Socioeconomic Disadvantage (IRSD) [50]. The IRSD scores were calculated using 2006 census data and derived by the ABS using Principal Components Analysis. A CCD’s IRSD score reflects each area’s overall level of disadvantage measured on the basis of 17 variables that capture a wide range of socioeconomic attributes, including; education, occupation, income, unemployment, household structure, and household tenure (plus others). For analysis, the 200 CCDs were grouped into quintiles based on their IRSD scores with Q1 denoting the 20% (n = 40) most disadvantaged areas in Brisbane and Q5 the least disadvantaged 20% (n = 40).

### Individual-level data, response rates, and measures

**Figure 1:**
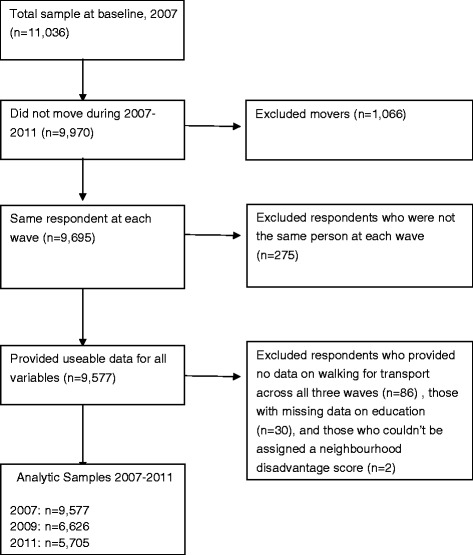
**Selecting the analytic samples.**

A structured self-administered questionnaire asked respondents about their neighbourhood, PA, sedentary behaviour, active transport, and correlates of these, and their socio-demographic characteristics. The questionnaire was administered in May 2007, 2009, and 2011 using a mail-survey method developed by Dillman [51]. After excluding out-of-scope respondents (i.e. deceased, no longer at the address, unable to participate for health-related reasons) the total number of useable surveys returned at each wave was 11,036, 7,867, and 6,901 respectively. The response rate at baseline was 68.4% (11,036 surveys from 16,128 eligible and contactable respondents); and 72.4% in 2009 (7,867/10,866) and 66.8% in 2011 (6,901/10,327). The baseline HABITAT sample was broadly representative of the wider Brisbane population, although residents from disadvantaged areas, blue-collar employees, and persons who did not attain a post-school educational qualification are underrepresented [49]. The analytic sample comprised respondents who lived at the same address for all three waves and provided useable data for all variables of interest (see Figure 1 for details of the analytic sample). The panel is unbalanced and allows for respondents to exit and re-enter the dataset irrespective of wave and item non-response.

### Measures

#### Education

Respondents were asked whether they had attained further education since leaving school, and if so, the highest qualification completed. Education was subsequently coded as (1) bachelor degree or higher (the latter included post graduate diploma, Masters, or doctorate) (2) diploma (associate or undergraduate), (3) vocational (trade or business certificate, or apprenticeship), (4) no post-school qualifications.

#### Employment status and occupation

Respondents reported their employment status at the time of the survey, and if employed, their job title and main tasks and duties performed. This information was coded in accordance with the Australian Standard Classification of Occupations (ASCO). ASCO is a skill-based measure that groups’ occupations according to levels of knowledge required, tools and equipment used, materials worked on, and goods and services produced. The occupational groupings are hierarchically ordered based on the relative skill-levels across these different dimensions, with those occupations having the most extensive skill requirements located at the top of the hierarchy. For analysis, the original nine-level ASCO was re-coded into 3 categories: (1) managers and professionals (managers and administrators, professionals and associate professionals); (2) white collar employees (clerical, sales and service); and (3) blue collar workers (trades, production workers, labourers). Three additional categories were created – (4) home duties, (5) retired, and (6) Not easily classified/missing, which included students, unemployed, permanently unable to work, and those who provided insufficient information for their employment status and/or occupation to be reliably classified.

#### Household income

Respondents were asked to estimate the total pre-tax income for their household using a single question comprising 13 income categories. For analysis, these were re-coded into six categories: (1) AUS$130,000 pa or more, (2) $129,999 – 72,800, (3) $72,799 – 52,000, (4) 51,999 – 26,000, (5) $25,999 – 0 and (6) Missing (i.e. left the income question blank, ticked ‘Don’t know’ or ‘Don’t want to answer this’).

#### Walking for transport (WfT)

At each wave this was identically measured using a single question that asked respondents to report how much time (minutes) they had spent walking for transport in the previous week (i.e., travel to and from work, to do errands, or to go from place to place). The distribution of the WfT variable at each wave was right-skewed and included outlier values which were top-coded to 840 minutes (i.e. 2 hours walking each day). Exploratory analysis of the WfT variable indicated that the observations comprised two relatively discrete groups. Group one – hereafter defined as ‘never walkers’ - included respondents who reported no walking for all three waves, or for two waves (if they responded only twice), or for one wave (if they only responded once). Group two – hereafter defined as ‘walkers’ – included respondents who reported that they walked for transport for at least one wave.

### General analytic approach

**Figure 2:**
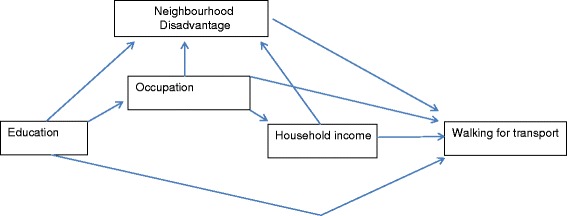
**Directed Acyclic Graph (DAG) conceptualising the relationships between neighbourhood disadvantage, individual-level SEP, and walking for transport.**

Previous research has found that indicators of SEP moderately correlate [52-54]; hence part of each measure’s association with health is shared with other socioeconomic indicators, and part of their contribution is unique. Shared variance arises because of the contextual and/or temporal relationships between neighbourhood disadvantage, education, occupation and income. For example, residents of disadvantaged neighbourhoods are on average likely to be less educated, employed in lower status occupations, and earning lower incomes than residents of advantaged neighbourhoods, thus giving rise to a correlation between neighbourhood disadvantage and individual-level SEP. Also, on average, educational attainment influences occupational outcomes, which in turn circumscribes income earning capacity, thus SEP earlier in the life-course is correlated with SEP over subsequent time periods. Given that socioeconomic measures share contextual and temporal variance, the use of a single indicator in an analysis of WfT will produce over-inflated model estimates for that indicator, because the included measure is capturing the impact of excluded socioeconomic measures. As a result, conclusions about the extent and nature of a measure’s influence on WfT may be over-stated. To assist with our analysis of this complexity we constructed a Directed Acyclic Graph (DAG) [55] which depicted the temporal ordering of education, occupation, and household income and the contextual influence of the neighbourhood environment (Figure 2), and this formed the basis for our analytic strategy addressing each question (see below).

### Analytic strategy for question one: who walks for transport?

Using the *reshape* command in Stata/SE Version 13 [56] a person-period data file was created which included a measure of time (2007, 2009, and 2011), and for each time-period, the dichotomised WfT variable (1=’never walker’; 0=’walker’), sex, age, neighbourhood disadvantage, education, occupation, and household income. We first undertook a descriptive analysis by examining the bivariate associations between non-walking, neighbourhood disadvantage, and each of the individual-level socioeconomic variables: the data are presented as the percentage of respondents who were classified as ‘never-walkers’ at each wave.

Second, guided by the DAG (Figure 2), we used a two-level mixed-effects logistic regression model to examine the multivariable association between neighbourhood disadvantage, individual SEP, and never-walking as follows.

#### Neighbourhood disadvantage

Differences between advantaged and disadvantaged neighbourhoods in the odds of never-walking were estimated with adjustment for within-neighbourhood variation in education, occupation, and household income.

#### Education

The association between never-walking and education was first estimated with adjustment for age, sex and year. The estimated odds ratios for education produced by this model were potentially imprecise as a result of confounding due to other unmeasured socioeconomic factors hence this initial model represented a base model against which more detailed models could be compared. The base model was subsequently extended by adjustment for neighbourhood disadvantage because in a previous paper we found that advantaged and disadvantaged neighbourhoods in Brisbane differed in the extent to which they facilitated WfT [57], and unpublished data from the HABITAT study showed that the percentage of low and high educated respondents is differently distributed across neighbourhoods varying in their level of socioeconomic disadvantage. Given these observations, failure to adjust the association between education and never-walking for neighbourhood disadvantage might overestimate the effects of education because this variable was partly capturing the unmeasured contextual influences of the neighbourhood environment. This model was further extended by adjustment for occupation and household income, because as the DAG postulates, these two socioeconomic factors represent part of the pathway via which education influences the likelihood of never-walking.

#### Occupation

For reasons similar to those above, the association between never-walking and occupation was firstly modelled with adjustment for age, sex, and year, then additionally for neighbourhood disadvantage, and then additionally for education and income.

#### Household income

The relationship between never-walking and household income was first modelled with adjustment for age, sex and year, then additionally for neighbourhood disadvantage, and then education and occupation.

The logistic regression models for the dichotomous WfT variable were fitted using MLwiN statistical software [58] and the model parameters were estimated using Markov chain Monte Carlo (MCMC) simulation with uninformative priors on all parameters in the model. All model results are reported as odds ratios (OR) with their 95% credible interval (CrI).

### Analytic strategy for question two: does walking for transport decline as we age?

In this analysis we are interested in how much walking for transport a respondent records if they do at least some walking and therefore we retained respondents who reported walking on at least one occasion, and removed those who were defined as ‘never-walkers’. The relationship between ageing and WfT was examined in MLwiN using a three-level mixed effects linear regression model with continuous measures for age (mean-centred at baseline), year (0 = 2007, 1 = 2009, 2 = 2011) and walking (minutes in the previous week). We initially present an unconditional (null) model comprising a fixed intercept (i.e. minutes of WfT at baseline) and three random terms that quantify the variation in WfT that is between-neighbourhoods (level 3), between-individuals (level 2), and within-individuals (level 1). This model is extended by adding fixed-effect terms for time, then age, and then a time-age interaction: this latter model assesses whether minutes of WfT change over time as we age. For all models we report the -2loglikelihood and compare model-fit using the deviance statistic.

### Analytic strategy for question three: is change in walking for transport associated with neighbourhood disadvantage and individual-level SEP?

For this analysis we used a person-period dataset comprising continuous measures of WfT, year, and age (mean-centred at each wave) and categorical measures for sex, neighbourhood disadvantage, education, occupation, and household income. We first conducted a descriptive analysis by examining bivariate associations between minutes of WfT in the previous week and each of the socioeconomic variables: these data are presented as mean minutes of walking (95% confidence interval) separately for each wave.

Second, we examined relationships between neighbourhood disadvantage, SEP, and change in WfT using the same analytic strategy as the logistic modelling. Model specifications were guided by the DAG (Figure 2); random terms quantified the variation in WfT that was between-neighbourhoods (level 3), between-individuals (level 2) and within-individuals (level 1); and each socioeconomic measure was interacted with time to assess whether change in walking as we age was socioeconomically patterned. For interactions that were statistically significant we plotted temporal trends in the association between neighbourhood disadvantage, SEP and WfT. For all models, the regression output is expressed as a parameter estimate that quantifies the absolute difference in minutes WfT relative to a reference group (i.e. least disadvantaged neighbourhoods, bachelor degree or higher, managers and professionals, $130,000pa or more). The parameter estimates are reported with 95% CI, which if not inclusive of zero, are considered to be significantly different from the reference group.

## Results

### Socioeconomic predictors of never-walking

The first three columns of Table 1 show that just under two-thirds of respondents at each wave were classified as a ‘never-walker’. The likelihood of never walking tended to be higher for residents of the least disadvantaged neighbourhoods, those with school-only education, blue collar workers, those engaged in home duties and the retired, and members of households earning $26,000 - $51,999 per annum.

**Table 1:** **Sample profile (2007–2011) of respondents defined as ‘never-walkers’, and mean minutes of walking for those defined as ‘walkers’** ^**1**^

	**Never-walkers** ^**2**^			**Minutes of walking** ^**3**^		
	**2007 (n = 9,488)**	**2009 (n = 6,392)**	**2011 (n = 5,609)**	**2007 (n = 4,666)**	**2009 (n = 3,595)**	**2011 (n = 3,284)**
	**%**	**%**	**%**	**Mean (95% CI)**	**Mean (95% CI)**	**Mean (95% CI)**
**Overall**	65.3	61.9	62.0	70.4 (67.2 – 73.6)	64.6 (61.0 - 68.2)	60.4 (57.1-63.8)
**Neighborhood disadvantage**						
Q5 (Least disadvantaged)	68.2	64.2	65.1	63.9 (57.8-70.0)	56.8 (50.6-63.1)	54.3 (48.4-60.1)
Q4	66.1	63.6	62.9	66.0 (59.8-72.3)	63.0 (55.9-70.2)	63.2 (55.7-70.7)
Q3	65.3	61.2	61.7	71.4 (64.0-78.8)	62.5 (54.6-70.5)	59.1 (51.9-66.4)
Q2	62.3	58.7	57.3	74.4 (66.9-81.9)	67.1 (59.0-75.2)	65.6 (57.5-73.8)
Q1 (Most disadvantaged)	61.9	59.2	60.2	82.1 (72.9-91.4)	81.1 (69.3-93.0)	62.4 (53.0-71.8)
**Highest attained education**						
Bachelor’s degree or higher	56.6	52.9	53.4	72.8 (67.6-77.9)	66.0 (60.5-71.5)	64.9 (59.8-70.0)
Diploma/Associate diploma	63.6	61.8	60.5	70.8 (61.4-80.3)	61.3 (50.6-71.9)	57.3 (47.1-67.6)
Vocational (trade/business)	69.6	65.8	66.4	70.4 (62.2-78.6)	63.6 (54.8-72.4)	55.7 (49.0-62.4)
School	70.6	67.7	68.1	67.7 (62.3-73.0)	64.7 (58.3-71.0)	58.6 (52.2-65.1)
**Occupation**						
Managers & Professionals	61.7	56.7	56.8	68.4 (63.3-73.5)	64.1 (58.7-69.5)	68.1 (62.3-73.8)
White collar	65.1	60.6	61.2	67.4 (61.6-73.2)	67.9 (60.1-75.7)	58.0 (51.3-64.7)
Blue Collar	73.9	72.1	70.5	73.4 (63.1-83.7)	69.1 (55.1-83.2)	71.9 (58.6-85.1)
Home duties	71.1	68.1	74.3	54.1 (41.0-67.2)	49.9 (37.1-62.0)	51.0 (35.7-66.3)
Retired	68.9	64.7	66.3	58.2 (47.9-68.5)	57.2 (45.7-68.7)	37.9 (32.4-43.3)
Missing (includes NEC)	61.0	61.5	57.0	88.5 (79.0-97.9)	68.8 (60.3-77.3)	71.0 (59.6-82.3)
**Household income**						
$130,000 pa or more	64.3	59.5	56.7	69.3 (61.5-77.2)	60.8 (54.3-67.3)	68.8 (61.2-76.3)
$72,800 - $129,999	63.8	58.4	60.7	68.6 (62.8-74.3)	65.7 (59.3-72.1)	60.4 (54.3-66.4)
$52,000 - $72,799	63.4	63.3	63.0	67.5 (60.2-74.5)	60.8 (51.2-70.4)	53.3 (44.6-62.0)
$26,000 - $51,999	67.5	64.9	65.4	72.1 (64.4-79.7)	61.4 (52.9-69.9)	57.1 (48.6-65.5)
$0 - $25,999	62.0	63.2	59.7	84.1 (71.5-96.7)	70.0 (57.3-82.7)	63.0 (50.9-75.1)
Missing	70.2	64.9	67.8	66.4 (57.6-75.3)	71.2 (59.2-83.2)	55.9 (47.5-64.4)

^1^Bivariate results, unadjusted for any other factors.

^2^Respondents who indicated no walking for transport for all three waves, or for two waves (if they only responded twice), or for one wave (if they only responded once).

^3^Respondents who reportedly walked for transport for at least one wave.

In Table 2 we present models examining associations between neighbourhood disadvantage, SEP, and the odds of never walking. In the first panel, we present the results for neighbourhood disadvantage. After adjustment for year, age, sex, education, occupation, and household income, a clear inverse association was observed between neighbourhood disadvantage and never walking, with the odds being lowest for residents of the most disadvantaged areas (OR 0.66, 95%CrI 0.49-0.88).

**Table 2:** **Neighbourhood disadvantage, individual-level socioeconomic position and the likelihood of not walking for transport** ^**1**^

**N = 21,489 observations**	**Model 1**		**Model 2**		**Model 3**	
	**OR**	**95%CrI**	**OR**	**95%CrI**	**OR**	**95%CrI**
**Neighbourhood disadvantage** ^**2**^						
Q5 (Least disadvantaged)	1.00					
Q4	0.94	0.72,1.22				
Q3	0.86	0.66,1.14				
Q2	0.73	0.53,0.97				
Q1 (Most disadvantaged)	0.66	0.49,0.88				
Between-neighbourhood variance (se)	0.371 (0.047)					
**Highest attained education** ^**3**^						
Bachelor’s degree or higher	1.00		1.00		1.00	
Diploma/Associate diploma	1.35	1.22,1.49	1.36	1.23,1.49	1.35	1.23,1.50
Vocational (trade/business)	1.75	1.61,1.91	1.77	1.62,1.93	1.68	1.53,1.84
School	1.93	1.79,2.06	1.94	1.81,2.09	1.90	1.75,2.06
Between-neighbourhood variance (se)	0.392 (0.048)		0.373 (0.047)		0.371 (0.047)	
**Occupation** ^**4,6**^						
Managers & Professionals	1.00		1.00		1.00	
White collar	1.15	1.06,1.24	1.15	1.06,1.25	0.88	0.81,0.96
Blue Collar	1.89	1.72,2.08	1.91	1.73,2.10	1.42	1.28,1.58
Home duties	1.38	1.21,1.57	1.38	1.21,1.58	1.03	0.89,1.18
Retired	1.01	0.91,1.13	1.01	0.91,1.13	0.82	0.74,0.92
Between-neighbourhood variance (se)	0.403 (0.049)		0.392 (0.049)		0.371 (0.047)	
**Household income** ^**5,6**^						
$130,000 pa or more	1.00		1.00		1.00	
$72,800 - $129,999	1.00	0.91,1.09	1.00	0.92,1.09	0.91	0.83,0.99
$52,000 - $72,799	1.06	0.95,1.17	1.07	0.96,1.18	0.92	0.83,1.03
$26,000 - $51,999	1.23	1.11,1.36	1.24	1.12,1.37	1.02	0.92,1.13
$0 - $25,999	1.07	0.95,1.21	1.08	0.96,1.22	0.91	0.80,1.03
Between-neighbourhood variance (se)	0.416 (0.050)		0.412 (0.051)		0.371 (0.047)	

^1^Defined as participants who reportedly didn’t walk for transport at each wave they responded to.

^2^Neighbourhood disadvantage adjusted for education, occupation, household income, age, sex, and year.

^3^Education adjusted for age, sex and year (Model 1), plus neighbourhood disadvantage (Model 2), plus occupation and household income (Model 3).

^4^Occupation adjusted for age, sex and year (Model 1), plus neighbourhood disadvantage (Model 2), plus education and household income (Model 3).

^5^Household income adjusted for age, sex and year (Model 1), plus neighbourhood disadvantage (Model 2), plus education and occupation (Model 3).

^6^The missing categories for occupation and household income were included in the statistical analysis but are not presented in the table.

In the second panel of Table 2 we present the results for education. The likelihood of never walking was significantly higher for people without a Bachelor’s degree (Model 1) and the magnitude of this association remained largely unchanged after adjustment for neighbourhood disadvantage (Model 2) and occupation and household income (Model 3).

The results for occupation are presented in the third panel of Table 2. The odds of never walking were significantly higher for white (OR 1.15, 95%CrI 1.06-1.24) and blue collar workers (OR 1.89, 95%CrI 1.72-2.08) and those engaged in home duties (OR 1.38, 95%CrI 1.21-1.57) relative to managers and professionals. These effects were of a similar magnitude before (Model 1) and after adjustment for neighbourhood disadvantage (Model 2). After further adjustment for education and household income (Model 3) the odds of never walking remained significantly higher for blue collar workers (OR 1.42, 95%CrI 1.28-1.58), became significantly lower for white collar workers (OR 0.88, 95%CrI 0.81-0.96) and the retired (OR 0.82, 95%CrI 0.74-0.92), and was attenuated to non-significance for home duties (OR 1.03, 95%CrI 0.89-1.18).

In the final panel of Table 2 we present the results for household income. There was no clear pattern of association between household income and never walking, either before (Model 1) or after adjustment for neighbourhood disadvantage (Model 2), although the odds were significantly higher for residents of households earning between Aus$26,000-$51,999 per annum. After further adjustment for occupation and education (Model 3) this relationship was attenuated to non-significance (OR 1.02, 95%CrI 0.92-1.13), and we now observed a statistically significant difference between the highest and second highest income groups in their odds of never walking, with the likelihood being approximately 9% lower in the latter income category (OR 0.91, 95%CrI 0.83-0.99).

In a null model (not presented in Table 2) the between-neighbourhood variance was statistically significant (U_0j_ 0.408, se 0.050) suggesting that the likelihood of never walking varied non-randomly across Brisbane’s neighbourhoods. The variance terms presented in Table 2 indicate that the neighbourhood differences in never walking observed in the null model were little affected by adjustment for neighbourhood disadvantage and within-neighbourhood variation in education, occupation and household income.

### Ageing and minutes walking for transport

Table 3 presents the association between time, age, and minutes WfT for respondents who reported walking on at least one occasion over the three waves. Between 2007 and 2011, Brisbane residents aged 40 – 70 years walked an average of 67.8 minutes for transport-related purposes in the previous week (Model 1). The between-neighbourhood random effect summarizes the extent of neighbourhood variability around this population average; the between-individual variance quantifies the unexplained differences between respondents in their average minutes of walking; and the within-individual random term summarizes each person’s temporal variability in minutes of WfT over the five-year reference period. Each of the random effects was significant at the 5% level and the variance components expressed as proportions of the total variance in WfT were 1.2%, 20.4% and 78.4% for between-neighbourhoods, between-individuals, and within-individuals respectively.

**Table 3:** **Minutes walking for transport by time and age, 2007–2011: random intercept models**

**N = 11,545 observations**	**Model 1**	**Model 2**	**Model 3**	**Model 4**
**Fixed effects** ^**1**^				
*Intercept(se)*	67.8(1.4)	71.0(1.7)	70.9(1.7)	71.0(1.7)
*Time (0 = 2007)*		−3.72(−5.9, −1.6)^‡^	−3.77(−5.9, −1.6)^‡^	−3.76(−5.9, −1.6)^‡^
*Age (centred)* ^2^			−0.63(−0.9, −0.3)^‡^	−0.36(−0.8, 0.1)^ns^
Age * Time interaction				−0.32(−0.6, 0.0)*
**Random effects** ^**1**^				
Between-neighbourhood variance (se)	131.8(39.8)	130.5(39.6)	135.3(40.0)	134.8(40.0)
Between-individual variance (se)	2336.2(139.6)	2313.1(139.2)	2295.2(138.8)	2302.6(138.8)
Within individual variance (se)	8969.1(150.9)	8975.1(150.9)	8971.7(150.9)	8962.8(150.7)
−2Log Likelihood	140206.8	140195.2^‡^	140180.3^‡^	140176.0*

^1‡^<0.001, *< 0.05, ^ns^= not statistically significant.

^2^Mean centred at baseline (2007).

**Figure 3:**
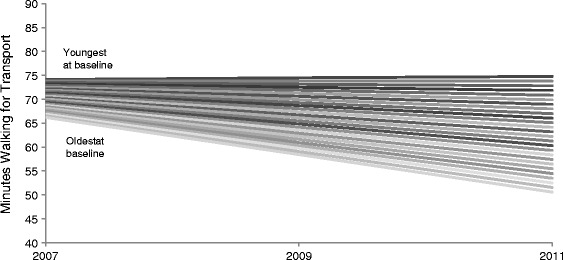
**Plotting the association between minutes of walking for transport in the previous week and age and time** ^**1**^ **.** ^1^Age at baseline (2007) ranged from 40 – 65 years.

Model 2 shows that in 2007 respondents were walking an average of 71.0 minutes per week, and that this declined by an average 3.7 minutes per wave (95% CI −5.9, −1.6) between 2007–2009 and 2009–2011. In Model 3, age was negatively associated with WfT: each additional year of age was associated with 0.63 fewer minutes of WfT each week. In Model 4 the time-age interaction was statistically significant indicating that as respondents aged they engaged in fewer minutes of WfT each week: this is graphically represented in Figure 3. Average minutes of walking each week remained relatively stable between 2007 and 2011 for persons aged 40 – 45 at baseline; however, for each successive increase in age beyond 45 years, average minutes of walking declined, with the declines being steeper among those aged 60 years or older at baseline. Age-differences in WfT also became increasingly heterogeneous over time.

### Neighbourhood disadvantage, individual-level SEP and change in minutes walking for transport

The last three columns of Table 1 present the average minutes of WfT in the previous week in 2007, 2009 and 2011 for respondents who reported walking on at least one occasion over the three waves. Average (overall) minutes of walking declined between 2007 and 2011 from 70.4 minutes per week in the former period to 60.4 minutes per week in the latter. At each wave, average minutes of WfT were higher for residents of disadvantaged neighbourhoods, those with a bachelor degree or higher, blue collar workers, and residents of the lowest income households (except in 2011). Average minutes of WfT were noticeably lower among those classified as home duties and the retired.

Table 4 (top panel) presents results for neighbourhood disadvantage and WfT. After adjustment for sex, age, education, occupation, and household income, there was a graded (linear) association between neighbourhood disadvantage and WfT: average minutes of walking was lowest among residents of the least disadvantaged neighbourhoods (63.4 minutes), intermediate among residents of neighbourhoods in quintile 3 (71.7 minutes), and highest among residents of the most disadvantaged neighbourhoods (80.9 minutes). The interaction between neighbourhood disadvantage and time was non-significant, indicating that average change (decline) in WfT between 2007 and 2011 was broadly similar for residents of neighbourhoods in each quintile of socioeconomic disadvantage.

**Table 4:** **Minutes walking for transport in the previous week by neighbourhood disadvantage (top panel) and education (bottom panel): main effect and time-interaction model**

**Neighbourhood disadvantage** ^**1**^	**Main effect**	**Interaction**
Intercept (se)	63.4 (3.9)	63.5 (4.2)
Q5 (Least disadvantaged)	--	--
Q4	6.5 (−1.2,14.1)^ns^	3.3 (−6.0,12.6)^ns^
Q3	8.3 (0.3,16.3)^*^	8.7 (−1.1,18.4)^ns^
Q2	11.0 (2.8,19.3)^†^	10.8 (0.7,20.8)^*^
Q1 (Most disadvantaged)	17.5 (8.7,26.2)^‡^	21.1 (10.5,31.7)^‡^
Q5 * time	--	--
Q4 * time		3.6 (−2.5,9.8)^ns^
Q3 * time		−0.4 (−6.9,6.0)^ns^
Q2 * time		0.3 (−6.3,6.9)^ns^
Q1 * time		−4.4 (−11.4,2.7)^ns^
Between-neighbourhood variance (se)	82.2 (33.8)	82.9 (33.8)
Between-individual variance (se)	2248.5 (137.6)	2253.5 (137.6)
Within individual variance (se)	8960.7 (150.7)	8952.4 (150.5)
−2Log Likelihood	140105.0	140100.2^ns^
**Education** ^**2**^	**Main effect**	**Interaction**
Intercept (se)	69.4 (2.5)	68.7 (2.8)
Bachelor’s degree or higher	--	--
Diploma/Associate diploma	−2.9 (−10.4,4.6)^ns^	−1.2 (−10.8, 8.5)^ns^
Vocational (trade/business)	−3.4 (−10.1,3.4)^ns^	−0.8 (−9.5, 7.8)^ns^
School	−2.0 (−7.4,3.5)^ns^	−1.7 (−8.6, 5.2)^ns^
Bachelor’s degree or higher * time		--
Diploma/Associate diploma * time		−2.0 (−9.1, 5.0)^ns^
Vocational (trade/business) * time		−2.9 (−9.3, 3.5)^ns^
School * time		−0.3 (−5.4, 4.8)^ns^
Between-neighbourhood variance (se)	133.0 (39.7)	133.0 (39.7)
Between-individual variance (se)	2278.2 (138.4)	2277.2 (138.4)
Within individual variance (se)	8975.2 (150.9)	8975.0 (150.9)
−2Log Likelihood^2^	140170.1	140169.0^ns^

^1^Neighbourhood disadvantage adjusted for education, occupation, household income, age, sex, and year.

^2^Education adjusted for age, sex, and year. The results remained unchanged after adjustment for neighbourhood disadvantage, and then further adjustment for occupation and household income.

^‡^<0.001, ^†^<0.01, *< 0.05, ns = not statistically significant.

Table 4 (bottom panel) presents results for education and WfT. There was no association between education level and minutes of WfT: although respondents with lower levels of education walked for fewer minutes in the previous week than their counterparts with a bachelor degree or higher, the differences were not statistically significant after adjustment for age and sex. These results remained unchanged after further adjustment for neighbourhood disadvantage, and then occupation and household income (results not shown). In addition, there was no significant interaction between education, time, and WfT: between 2007 and 2011 respondents in each education group showed a similar rate of decline in the number of minutes spent WfT (results not shown).

**Figure 4:**
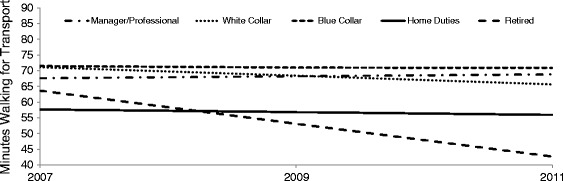
**Plotting the association between minutes walking for transport in the previous week and occupation: 2007 - 2011** ^**1**^ **.** ^1^The plot was produced using the regression estimates from Model 3 (interaction) in Table 5.

Table 5 presents results for occupation and minutes WfT. Among the employed, there was no association between occupation and WfT: average number of minutes of WfT was similar for managers and professionals, white collar employees, and blue collar workers, and this pattern was observed before (Model 1) and after adjustment for other socioeconomic factors (Models 2 & 3). Moreover, there was no significant interaction between occupation, time, and WfT: between 2007 and 2011 the number of minutes of walking reported by respondents in each occupation category remained relatively stable. The results in Table 5 indicate that respondents outside of the paid workforce had a somewhat different walking profile than their employed counterparts. Compared with managers and professionals, respondents who nominated ‘home duties’ or ‘retired’ as their primary daily role walked an average of 11 (95% CI −21.2, −0.9) and 15 (95% CI −22.2, −6.9) minutes less in the previous week respectively (Model 1). These findings were largely unchanged after adjustment for neighbourhood disadvantage (Model 2) and education and household income (Model 3). There was no significant interaction between home duties, time, and WfT: for these respondents, average minutes of WfT remained largely unchanged between 2007 and 2011 (Figure 4). By contrast, average minutes of WfT among the retired declined markedly over the 5 year reference period: for the fully adjusted analyses, retired respondents in 2007 reported walking an average of 63 minutes in the previous week, 53 minutes in 2009, and 42 minutes in 2011.

**Table 5:** **Minutes walking for transport in the previous week by occupation: main effect and time-interaction models**

	**Model 1**		**Model 2**		**Model 3**	
**Occupation** ^**1,2**^	**Main effect**	**Interaction**	**Main effect**	**Interaction**	**Main effect**	**Interaction**
Intercept (se)	68.3 (2.6)	65.0 (2.9)	60.9 (3.4)	57.7 (3.6)	63.4 (3.9)	60.1 (4.1)
Managers & Professionals	--	--	--	--	--	--
White collar	−0.1 (−6.0, 5.8)^ns^	2.8 (−4.9, 10.6)^ns^	−1.2 (−7.1, 4.8)^ns^	1.8 (−5.9, 9.6)^ns^	0.6 (−5.8, 7.0)^ns^	3.5 (−4.6, 11.6)^ns^
Blue Collar	2.3 (−5.2, 9.9)^ns^	3.3 (−6.5, 13.1)^ns^	0.7 (−6.9, 8.3)^ns^	1.7 (−8.2, 11.5)^ns^	3.0 (−5.2, 11.1)^ns^	3.7 (−6.5, 14.0)^ns^
Home duties	−11.0 (−21.2, −0.9)*	−9.5 (−23.1, 4.2)^ns^	−12.0 (−22.1, −1.9)*	−10.7 (−24.3, 3.0)^ns^	−11.3 (−21.8, −0.8)*	−10.1 (−24.0, 3.8)^ns^
Retired	−14.6 (−22.2, −6.9)^†^	−2.2 (−13.2, 8.8)^ns^	−15.7 (−23.3, −8.0)^†^	−3.4 (−14.4, 7.7)^ns^	−16.0 (−24.0, −7.9)^†^	−3.9 (−15.2, 7.4)^ns^
Managers & Professionals * time		--		--		--
White collar * time		−3.5 (−9.5, 2.6)^ns^		−3.5 (−9.6, 2.6)^ns^		−3.4 (−9.5, 2.7)^ns^
Blue Collar * time		−1.1 (−8.8, 6.6)^ns^		−1.1 (−8.8, 6.7)^ns^		−0.9 (−8.6, 6.9)^ns^
Home duties * time		−1.8 (−12.7, 9.2)^ns^		−1.5 (−12.4, 9.5)^ns^		−1.3 (−12.3, 9.6)^ns^
Retired * time		−11.4 (−18.5, −4.3)^†^		−11.3 (−18.5, −4.2)^†^		−11.1 (−18.3, −4.0)^†^
Between-neighbourhood variance (se)	128.7 (39.1)	128.3 (39.0)	89.7 (34.7)	90.2 (34.7)	82.2 (33.8)	82.6 (33.8)
Between-individual variance (se)	2258.4 (137.8)	2265.9 (137.8)	2262.6 (137.9)	2268.7 (137.8)	2248.5 (137.6)	2254.9 (137.6)
Within individual variance (se)	8957.2 (150.6)	8939.6 (150.3)	8957.5 (150.6)	8940.1 (150.3)	8960.7 (150.7)	8943.6 (150.4)
−2Log Likelihood	140133.2	140119.0^†^	140116.4	140102.4^†^	140105.0	140091.5^†^

^1^Occupation adjusted for age, sex, and year (Model 1), plus neighbourhood disadvantage (Model 2), plus education and household income (Model 3).

^2^The missing category for occupation was included in the statistical analysis but not presented in the table.

^†^<0.01, *< 0.05, ^ns^= not statistically significant.

**Figure 5:**
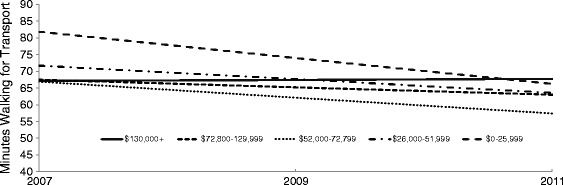
**Plotting the association between minutes walking for transport in the previous week and household income: 2007-2011** ^**1**^ **.** ^1^The plot was produced using the regression estimates from Model 3 (interaction) in Table 6.

Table 6 presents associations between household income and minutes WfT. After adjustment for age and sex (Model 1), residents of the lowest income households walked an average 9 (95% CI 0.6, 17.4) minutes more per week than residents of the most affluent households: this difference however was attenuated and became non-significant after further adjustment for neighbourhood disadvantage (Model 2) and then education and occupation (Model 3). The results from interacting household income with time show that minutes spent WfT declined for most income groups between 2007 and 2011: the decline was steepest for residents of the lowest income households (Figure 5).

**Table 6:** **Household income and minutes walking for transport in the previous week: main effect and time-interaction models**

	**Model 1**		**Model 2**		**Model 3**	
**Household income** ^**1,2**^	**Main effect**	**Interaction**	**Main effect**	**Interaction**	**Main effect**	**Interaction**
Intercept (se)	67.3 (3.1)	63.5 (3.8)	61.1 (3.7)	57.4 (4.3)	63.4 (3.8)	60.3 (4.4)
$130,000 pa or more	--	--	--	--	--	--
$72,800 - $129,999	−1.2 (−7.3, 4.9)^ns^	1.6 (−7.0, 10.2)^ns^	−2.6 (−8.7, 3.6)^ns^	0.2 (−8.4, 8.8)^ns^	−2.1 (−8.3, 4.4)^ns^	0.5 (−8.2, 9.1)^ns^
$52,000 - $72,799	−3.6 (−10.8, 3.7)^ns^	1.6 (−8.3, 11.5)^ns^	−5.7 (−13.1, 1.6)^ns^	−0.5 (−10.4, 9.5)^ns^	−4.6 (−12.1, 2.8)^ns^	−0.1 (−10.0, 10.0)^ns^
$26,000 - $51,999	1.2 (−5.9, 8.4)^ns^	6.5 (−3.2, 16.2)^ns^	−1.4 (−8.6, 5.9)^ns^	3.7 (−6.1, 13.5)^ns^	−0.8 (−6.8, 8.3)^ns^	4.8 (−5.2, 14.8)^ns^
$0 - $25,999	9.0 (0.6, 17.4)*	17.8 (6.4, 29.2)^†^	5.2 (−3.4, 13.8)^ns^	13.8 (2.3, 25.4)^†^	7.6 (−1.4, 16.6)^ns^	15.1 (3.2, 26.9)^†^
$130,000 pa or more * time		--		--		--
$72,800 - $129,999 * time		−2.8 (−9.5, 3.9)^ns^		−2.8 (−9.4, 4.0)^ns^		−2.6 (−9.3, 4.0)^ns^
$52,000 - $72,799 * time		−5.8 (−13.6, 2.1)^ns^		−5.8 (−13.7, 2.0)^ns^		−5.2 (−13.1, 2.7)^ns^
$26,000 - $51,999 * time		−5.8 (−13.1, 1.5)^ns^		−5.6 (−12.8, 1.7)^ns^		−4.4 (−11.7, 2.9)^ns^
$0 - $25,999 * time		−9.8 (−18.3, −1.2)*		−9.5 (−18.1, −1.0)*		−8.3 (−16.8, 0.3)^ns^
Between-neighbourhood variance (se)	125.7 (38.9)	124.1 (38.7)	90.3 (34.8)	89.3 (34.7)	82.1 (33.7)	89.3 (34.7)
Between-individual variance (se)	2270.4 (138.3)	2274.9 (138.3)	2274.8 (138.3)	2278.8 (138.3)	2248.5 (137.6)	2278.8 (138.3)
Within individual variance (se)	8976.6 (151.0)	8968.4 (150.8)	8976.0 (150.9)	8968.1 (150.8)	8960.7 (150.7)	8968.1 (150.8)
−2Log Likelihood	140162.1	140155.8^ns^	140146.3	140140.2^ns^	140105.0	140100.2^ns^

^1^Household income adjusted for age, sex, and year (Model 1), plus neighbourhood disadvantage (Model 2), plus occupation and education (Model 3).

^2^The missing category for household income was included in the statistical analysis but not presented in the table.

^†^<0.01, *< 0.05, ^ns^= not statistically significant.

### Random effects for minutes of walking for transport

The between-neighbourhood, between-individual, and within-individual variance terms are presented in the bottom rows of Tables 4, 5 and 6 and were significantly different from zero for all models. Together, these results indicate that (i) Brisbane neighbourhoods varied in the number of minutes that residents spent WfT relative to the average for Brisbane City overall, (ii) irrespective of where they lived, individuals varied widely in their reported minutes of WfT relative to the average number of minutes for the Brisbane population of mid-aged adults, and (iii) each individual respondent exhibited extensive heterogeneity in terms of how much they reported walking at each wave.

In terms of the age- and sex-adjusted models for education, occupation, and household income, within-individual variation accounted for the largest proportion of the total variance in WfT (~79% for the main effect models), followed by the between-individual (~20%) and between-neighbourhood variation (~1%). Subsequent adjustment of the main effect models for other socioeconomic factors accounted for between nil and a small amount of the between- and within-individual variance in WfT; however, the between-neighbourhood variance was reduced by an average of 46% (range 39%-57%) after adjustment for neighbourhood disadvantage.

## Discussion

This paper examined longitudinal relationships between neighbourhood disadvantage, individual-level SEP, and WfT and how these associations changed over time as people aged. An important reference-point for this research was studies that had examined the association between neighbourhood disadvantage, SEP, and LTPA. A large body of work extending back numerous decades consistently shows a positive association between socioeconomic circumstances and LTPA irrespective of how these concepts are measured [29,30]. By contrast, the relationship between neighbourhood disadvantage, individual-level SEP, and WfT has been investigated in a limited number of studies, all of which are cross-sectional, with mixed and inconsistent findings [27].

### Ageing and walking for transport

At baseline (2007) respondents were aged 40–65 years, and younger persons walked more minutes on average per week than their older counterparts. Between 2007 and 2011 average minutes of WfT remained fairly stable for younger respondents; however, for older persons, average minutes of walking declined markedly over the subsequent five years. These declines in WfT are consistent with findings from the PLACE study in Adelaide, Australia [59] and parallel those observed in longitudinal studies of LTPA [60,61]. Possibly, and especially for the older respondents in the HABITAT sample, age-related declines in WfT may be partly associated with concomitant declines in health and functional status [15].

Remaining physically active as we age is beneficial for aerobic capacity, strength, endurance, flexibility, range of motion, and balance control [2]. A reduced capacity or loss of these functions is often associated with reduced autonomy and independence, reduced community and family participation, and a lower quality of life; and for society and the economy, these flow on to increased health care costs and greater use of health- and aged-care systems, reduced rates of workforce participation and earlier retirement, and lower levels of civic engagement such as volunteering [62]. The challenge therefore is to find ways to delay the onset of the diseases and disabilities of ageing, thus enabling people to live longer in good health until death at an advanced old age. Meeting this policy challenge will require national, state, and local governments to configure (design and/or retrofit) neighbourhood environments to facilitate active transport and LTPA for an ageing population, and to develop sustainable individual-level interventions that can promote and support all domains of PA as we age.

### Neighbourhood disadvantage and walking for transport

Residents of disadvantaged neighbourhoods were significantly less likely to be classified as ‘never-walkers’: and among those who reportedly walked, residents of disadvantaged neighbourhoods spent significantly more time each week walking for transport-related purposes. Given that active transport is health protective independent of LTPA [39-45], greater minutes of walking among residents of disadvantaged areas may potentially minimise neighbourhood-level socioeconomic inequalities in total PA, and by extension, health inequalities for activity-related chronic disease such as CVD and diabetes.

These findings from Brisbane (Australia) are consistent with patterns of WfT reported by Giles-Corti and Donovan in Perth (Australia) [31] and Van Dyck et al. in Ghent (Belgium)[63]; however, they contrast with those reported by Cerin et al. in Adelaide (Australia)[33] who found no association between neighbourhood SES and weekly minutes of WfT, and a significant positive association between neighbourhood SES and weekly frequency of walking for transport. Methodological differences notwithstanding, these inconsistencies might point to variation between cities in terms of how urban infrastructure is spatially distributed along socioeconomic lines, thus differentially shaping and circumscribing socioeconomic patterns in WfT: national and international comparative research is needed to examine these issues.

The longitudinal evidence showed that minutes of WfT declined over time in all neighbourhoods, irrespective of the neighbourhood’s level of socioeconomic disadvantage. The declines appeared to be steeper for residents of the most disadvantaged neighbourhoods (figure not shown), although the interaction between neighbourhood disadvantage and time was not statistically significant. In Brisbane at least, disadvantaged neighbourhoods have physical environments that are more conducive to WfT [57]: they are typically more residentially dense, have a more interconnected street network, and a more diverse mix of land uses (hence a greater number of destinations within walking distance). In infrastructural terms therefore, disadvantaged neighbourhoods in Brisbane provide the ‘best’ contexts to facilitate and promote WfT: moreover, these environments may be important in offsetting the negative health consequences that often result from exposure to socioeconomic inequality at the individual-level. Even in these highly walkable neighbourhoods however, levels of walking declined over time as people aged. The reasons for these declines are as yet unknown; however, they represent a significant challenge to public health advocates in their attempts to keep people active and healthy in their later years of life. Further, given that WfT often takes place within the neighbourhood environment, declines in walking as we age are likely to translate to a lower level of direct and visible engagement with the local community, with potential negative flow-on effects for neighbourliness and social capital [64].

### Education level and walking for transport

Lower educated groups were significantly more likely to be classified as “never-walkers”; however, there were no education differences in minutes walked among those who reported walking. Moreover, there were no significant interactions between education, time, and WfT, thus levels of walking declined at a similar rate between 2007 and 2011 for all respondents irrespective of their level of education (figure not shown). At this stage we can only speculate about *why* low educated groups were less likely to walk for transport as very little research has investigated this issue. Higher educated groups tend to have a greater level of awareness of the links between motorised travel and environmental problems (e.g. pollution, greenhouse gas emissions) which could promote increased levels of WfT among this group [33]. Cerin et al. [33] also suggests that lower educated groups may be less positively pre-disposed to transport walking as they are less likely to perceive the health benefits of an active lifestyle, including participating in LTPA, which possibly reflects a lower responsiveness to health promotion messages. The findings of this present paper, and the interpretive evidence reported by Cerin et al. [33], need to be viewed circumspectly against a backdrop of a small number of studies that have examined the relationship between education and WfT, and which are highly variable in terms of how walking has been measured. Walking has been operationalised using indicators that capture any WfT (yes/no)[65], walking to work [47,66], walking to public transit [67], minutes WfT in general [38,63,67,68], walking for transport for ≥30 minutes per day [38,69], frequency of WfT [68], and WfT at a moderate or brisk pace [68]. This diverse body of work has produced mixed and sometimes inconsistent findings and generated a complex picture of the relationship between education and WfT that is not easily summarised: thus no clear trends or patterns can be reliably discerned. By extension, any efforts to better understand educational differences in WfT are arguably premature as the field is still someway from reaching a consensus about the form and direction of the relationship: replication studies are needed to provide a deeper evidence-base to advance knowledge and to more robustly inform policy and promotion efforts to increase WfT among all education groups.

### Occupation and walking for transport

The odds of never walking were significantly higher for white- and blue-collar workers and those engaged in home duties relative to managers and professionals. There were no differences between managers and professionals and retirees in the odds of never walking. Each of these relationships remained largely unchanged after adjustment for neighbourhood disadvantage; however, they changed markedly with further adjustment for education and household income. Specifically, the odds relative to managers and professionals became significantly lower for white collar workers and retirees; they remained significantly higher for blue collar workers, although substantially reduced; and they were attenuated to non-significance for home duties. A key message from these pre- and post-adjusted findings is the need to specify models and test relationships between socioeconomic variables in a theoretically informed way (e.g. via the use of DAGs) to avoid inaccurate population inferences and erroneous conclusions and policy recommendations.

Among those who walked for transport, differences in minutes walking in the previous week were observed between the employed and non-employed, but no differences were found between the occupation groups. Over the period 2007 to 2011, those classified as home duties and the retired, walked for transport approximately 11 minutes and 15 minutes less each week respectively than managers and professionals. Presumably, more minutes of walking among managers and professionals (and also among white- and blue-collar workers) reflected their travel to work which often involves the use of public transport. No known studies have examined the association between occupation and WfT, and the few that have investigated walking differences by employment status have produced mixed results. In contrast to this present study, Van Dyck et al’s study of Belgian adults aged 18–65 years [63] found that minutes of WfT were significantly higher among the non-employed. Similarly, Cole et al’s study of Australian adults aged 18 years and over [69] found no bivariate association between being in paid work (yes/no) and WfT for males, and a significant association for females, with employed females being more likely to report that they walked at a moderate or brisk pace. This same study however found no association between paid work and WfT for at least 150 minutes per week.

Our longitudinal results indicated that minutes spent WfT in the previous week remained relatively stable over the five-year study period for the occupation groups and those classified as home duties. Retired respondents by contrast exhibited a marked decline in average minutes of WfT between 2007 and 2011. The steeply downward trend for retirees possibly reflects a number of interacting factors including: a transition out of paid employment and hence less use of public transport and work-related walking [38]; older age; poorer health and functioning associated with ageing; increased fear and concerns about safety and crime in the neighbourhood [70] and therefore fewer walking trips to local destinations (e.g. shops, health care services); and a greater reliance on a motor vehicle.

### Household income and walking for transport

Household income did not strongly differentiate whether or not respondents WfT. Members of households earning between $26,000 and $51,999 had a significantly raised odds (23%) of never walking compared with members of the highest income households: no other income differences were found. This association remained unchanged after adjustment for neighbourhood disadvantage, but was attenuated to non-significance after further adjustment for education and occupation: this suggests that part of the association between income and not walking is due to the unmeasured influence of a respondent’s educational attainment and their occupational status.

Members of the lowest income households who were classified as walkers walked significantly more minutes per week than members of the highest income households. Similar findings have been consistently observed in other studies [33,38,47,66-68]. Limited access to a motor vehicle is posited as the main reason why members of low income households walk more for transport [33,57,68] although greater time constraints in high income households due to longer working hours [71] and a preference for PA during leisure time might also account for some of these differences in WfT [68].

In this present study, the relationship between household income and minutes of WfT was attenuated to non-significance after adjustment for neighbourhood disadvantage, thus the disproportionate concentration of low income households in disadvantaged neighbourhoods, and the more walkable environments of these neighbourhoods in Brisbane [57], partly accounted for the higher levels of WfT observed among members of low income households. Previous studies investigating the relationship between household income and WfT which didn’t adjust for neighbourhood disadvantage may therefore have over-estimated the extent to which income *per se* directly influenced one’s propensity to walk for transport-related purposes.

Between 2007 and 2011, minutes of WfT declined for all income groups; however, the declines were steeper for respondents from the lowest income households. During the later years of adulthood, members of poorer households typically experience higher rates of chronic degenerative disease and a greater loss of physical function than their more affluent counterparts [23,72], hence steeper declines in walking in low income households may reflect the greater burden of disease borne by this group. Poorer health and function in adulthood is more often experienced by those from disadvantaged backgrounds in childhood, and those who were exposed to more episodes of accumulated socioeconomic disadvantage over the life course [22]. Hence, the greatest gains in keeping older people physically active and healthy, and reducing health inequalities, are likely to result from policy investments that improve social, economic and environmental conditions in both early and later life.

### The random effects: their interpretation and implications

Mixed-effects linear regression models were used to examine associations between ageing and average minutes WfT, and change in WfT by neighbourhood disadvantage and individual-SEP. The random coefficients produced by these models offer useful insights into how average time spent WfT varies between neighbourhoods, between individuals, and within individuals, and what factors might contribute to this variation. The between-neighbourhood variation captured the extent to which average minutes of WfT in the 200 neighbourhoods varied around the overall average for Brisbane city. Before and after adjustment for age, sex, and each of the individual-level socioeconomic factors, the between-neighbourhood variation accounted for the smallest proportion of the total variance in WfT (~1% in each model), although all of the variance terms were significantly different from zero. Subsequent adjustment for neighbourhood disadvantage reduced the between-neighbourhood variance in average minutes of WfT by between 39% and 57%, reflecting the fact that advantaged and disadvantaged neighbourhoods in Brisbane differ in the extent to which their built environments are conducive to walking for transport-related purposes [57].

The between-individual variation summarises the extent to which average minutes of WfT for each of the sampled respondents varied around the overall average for the population of mid-aged adults living in Brisbane between 2007 and 2011. The between-individual variation in WfT accounted for approximately 20% of the total variation in WfT. When the null model (Model 1, Table 3) and all other models were compared, the magnitude of the between-individual variance was reduced by a maximum of 3.8%, thus age and sex and each of the socioeconomic factors accounted for only a small amount of the variation in WfT among Brisbane residents.

The within-individual variance captured the extent to which each individual varied over time in their reporting of WfT. Relative to the other two sources of variation, the within-individual variance was disproportionately large, accounting for approximately 79% of the total variance in WfT. The large within-individual variance suggested that there was very little temporal stability in peoples’ reporting of WfT: this was confirmed on examination of a sample of individual growth-plots which showed substantial between-wave heterogeneity in reported minutes of WfT. Asking respondents every two years to recall minutes of walking in the previous week appears problematic: a seven-day reference period is narrow and may not capture ‘usual’ activity making it difficult to detect systematic change. This has important implications for the conceptualisation and measurement of PA questions in longitudinal research, as questions that accurately elicit information about minutes of activity in cross-sectional studies won’t necessarily perform well in longitudinal studies [73]. It is therefore recommended that researchers direct attention to developing time-sensitive questions that are more ideally suited to reliably capturing change in PA, also being mindful of the time-lapse between data collection waves. In longitudinal studies it may be preferable to capture ‘usual’ behaviour rather than ‘last 7 days’, despite the tendency for the former behaviour to be over-reported.

### Study strengths and limitations

The study was based on a sample of Brisbane residents who lived at the same address between May 2007 and May 2011: delimiting the study to non-movers dampened the potential negative impact of neighbourhood self-selection, although this may have occurred prior to the baseline data being collected.

Our finding of an association between neighbourhood disadvantage and WfT might be confounded by individual-level socioeconomic factors not included in the models. However, we used the three most commonly employed individual-level indicators of SEP in health research (i.e. education, occupation, and income)[74], and given the correlation among these measures [54] it is likely they captured the unmeasured influence of other socioeconomic factors not included in the models.

An attrition analysis (not presented here) showed that the probability of loss to follow-up was significantly higher among younger respondents, the least educated, blue collar workers, members of low income households, and residents of disadvantaged neighbourhoods; however, the likelihood of drop-out was significantly lower among the retired, and those who reported walking for transport. Higher rates of loss to follow-up among the socioeconomically disadvantaged may bias the findings towards or away from the null depending on how the losses are associated with walking. Higher rates of attrition among the low SES that are non-differential with respect to WfT will typically only bias towards the null.

Walking for transport was measured by self-report using a question that asked respondents to estimate the total time they spent walking in the last week. Retrospective accounts of time-based activities are prone to substantial recall error [33,75]. Moreover, the extent and direction of recall error often varies by the respondents’ sociodemographic characteristics (e.g. young versus old, high versus low SES). Given this, the error inherent in our measure of weekly walking likely biased associations between neighbourhood disadvantage, individual-level SEP and WfT, although it is not known if the bias resulted in an under- or over-estimation of the effect-sizes relative to their ‘true’ magnitude in the wider population.

The measure of walking was non-specific in its focus hence peoples’ reporting probably captured a diverse range of travel-related activities such as use of public transport for employment, taking children to school, and accessing businesses and services in the local neighbourhood. Associations between neighbourhood disadvantage, individual-level SEP and WfT appear to vary depending on the purpose of the walking and the destination (as noted above). The findings of this study therefore may have shown a different patterning and magnitude with a closer conceptual alignment between the socioeconomic predictor and the walking activity (e.g. full-time home duties and walking children to school, occupation and employment status and walking to public transport, retired and use of health care services). Future research should investigate this issue further as it is consistent with recent calls for greater specificity in our conceptualisation, measurement, and modelling of the determinants of PA, including walking for transport [76]. Further, the measure of walking provided no indication of the exertion-level or intensity of the activity. Walking at a moderate pace is deemed necessary to produce health benefits [69] and as this study didn’t capture this we were unable to determine if respondents were meeting PA recommendations by walking for transport. This said however, WfT for any intensity or duration is likely to accrue some health benefits relative to no activity [38].

Finally, for a range of reasons, comparing the findings of this present study with earlier research was difficult. Existing studies have measured WfT in a variety of ways (e.g. categorical or continuous, frequency, minutes, or intensity) using different scenarios (e.g. walking to places in general or to specific destinations such as public transport). Studies used different reference periods when asking about WfT (e.g. daily, weekly, or fortnightly), the analytic models were specified using different types and numbers of socioeconomic indicators and covariates (e.g. self-rated health, body mass index), and the residential contexts in which the studies have been conducted possibly differed on neighbourhood-level factors likely to influence WfT such as residential density, land use mix, street connectivity, and closeness to walkable destinations (e.g. shops, employment, transport nodes). This present study investigated WfT using a sample aged 40–65 in 2007 (baseline), whereas others used either an all-ages sample [37,66], a sample that included respondents who were 18 years or older [38,67,69], or a sample where respondents were aged between 18 and 65 years [31,33,47,63,65,68]. Clearly, the lack of consistency across studies in their designs, samples, methods, measures, and reference periods makes it difficult to reliably compare findings; it also arguably thwarts efforts to further advance our understanding of how and why neighbourhood disadvantage and individual-level SEP are related to WfT.

## Conclusion

Walking for transport declines as we age, irrespective of one’s socioeconomic background; however, the rate of decline is steeper for some socioeconomically disadvantaged groups, possibly as a result of their poorer health and functioning in mid-age and older adulthood. Given that WfT is health-protective, keeping people active as they age, especially those from more disadvantaged socioeconomic circumstances, will require the simultaneous implementation of policies that are directed at neighbourhoods and their residents.

Associations between neighbourhood disadvantage, individual-level SEP and WfT show little consistency in direction; rather, associations vary depending on the level of socioeconomic measurement, the type of individual-level socioeconomic indicator used, and how WfT is measured. The conditionality of the associations suggests that different explanatory mechanisms and processes may be encapsulated in the causal pathway linking each socioeconomic marker with WfT. If future research confirms this, then we are likely to need a mix of universal and targeted intervention strategies to increase WfT rather than a one-size fits-all approach [33].
